# 

**Published:** 1845-02

**Authors:** 


### CONTENTS

OF NO. II.

---

### ORIGINAL COMMUNICATIONS.

### ESSAYS AND CASES.

ART. I.—An account of Putrid Sore Throat, as it occurred in some parts of Ohio. By JOHN DAWSON, M.D., of Jamestown, Ohio. - - - - - - - 93

### REVIEWS.

ART. II.—Lectures on the Theory and Practice of Physic. By JOHN BELL, M.D., Fellow of the College of Physicians of Philadelphia; Corresponding Secretary of the Philadelphia Medical College; Member of the American Philosophical Society, and of the Georgofili Society of Florence: and by WILLIAM STOKES, M.D., Lecturer at the Medical School, Park Street, Dublin; Physician to the Meath Hospital, &c., &c. Third edition, enlarged and improved. In 2 vols. - 138

### SELECTIONS FROM AMERICAN AND FOREIGN JOURNALS

The Abortive and Curative Treatment of Gonorrhœa by the Nitrate of Silver, with Cases. By H. F. Campbell. - 153
Accidental Poisoning with Arsenic successfully treated with the Hydrated Peroxide of Iron. By J. J. Kelso. - 156

Efficacy of Calomel in large doses in Typhus Fever aided by Cold Affusions. By Joshua Burgess. - - - 161
Diseases of the Negro Population. By Daniel Drake. - 164
An Improved Mode of Managing Blisters. By John Key Robertson. - - - - - - - - - - 167
Extirpation of the Uterus for Complete Inversion. - - 168
Charges for Homœopathic Services. - - - - - - 168
Treatment of Pleurisy. - - - - - - - - 172
Cure of Nævi by Croton Oil. - - - - - - - 172
Report of Cases of Scarlatina, having relation to the question of the contagiousness of that diseases. By J. K. Mitchell. - - - - - - - - - - - 173
Treatment of Hydrocele with Ioduretted Injections. - - 175
Death of Dr. Abercrombie. - - - - - - 175
Succinate of Ammonia—a remedy for Delirium Tremens. - 178

ORIGINAL INTELLIGENCE.

Intermittent, Remittent, and Congestive Fevers, - - 179
Medical Examiner, - - - - - - - - - 180
Epidemic Erysipelas, - - - - - - - - - 180
Rate of Mortality among the Students of the Medical Institute of Louisville, - - - - - - - - - 181
Sixth Annual Report of the Ohio Lunatic Asylum, - - 183
Prize Essays, - - - - - - - - - - - 184

